# *Taenia multiceps* coenurosis: a review

**DOI:** 10.1186/s13071-022-05210-0

**Published:** 2022-03-12

**Authors:** Antonio Varcasia, Claudia Tamponi, Fahad Ahmed, Maria Grazia Cappai, Francesca Porcu, Naunain Mehmood, Giorgia Dessì, Antonio Scala

**Affiliations:** 1 Department of Veterinary Medicine, Veterinary Teaching Hospital, University of Sassari, Sassari, Italy; 2 Department of Zoology, University of Sargodha, Sargodha, Pakistan

**Keywords:** *Taenia multiceps*, Coenurosis, Cestodes, Sheep, Goats, Dog, Fox, Zoonosis

## Abstract

*Taenia multiceps* is a taeniid cestode that inhabits the small intestines of both wild and domestic carnivores. The larval stage, *Coenurus cerebralis*, is typically found in the central nervous system (CNS) of a wide range of livestock and, to a lesser extent, in the extra-cerebral tissues of sheep and goats. This review covers all aspects of the life cycle of *T. multiceps* and its epidemiology, molecular characterization, pathogenesis, diagnosis, therapy, control and zoonotic potential. Coenurosis caused by the larval stage of *T. multiceps* has a worldwide distribution and is often fatal in intermediate hosts, which can result in substantial economic losses in livestock farming. Molecular characterization using the mitochondrial genes cytochrome c oxidase subunit 1 and nicotinamide adenine dinucleotide dehydrogenase subunit 1 of different *T. multiceps* populations has revealed significant genetic variation and the presence of three major haplotypes. The disease mostly affects young sheep and is referred to as either acute or chronic coenurosis. Acute coenurosis occurs as a result of oncospheres migrating through the CNS, while chronic coenurosis occurs as a consequence of the coenurus maturing, which causes displacement and pressure atrophy of brain tissue. Non-cerebral coenurosis has been most commonly reported in goats. The best diagnostic method for cerebral coenurosis involves the interpretation of clinical signs with accurate localization of the cyst using diagnostic imaging techniques. A vaccine based on recombinant oncosphere antigens has proved to be an effective tool against *T. multiceps* infection in sheep. Additionally, use of anthelmintics during the parasite’s migration stages reduces the development of cysts in the sheep brain. Surgery is considered the most effective method for the treatment of cerebral coenurosis in small ruminants, but is often not carried out because of the limited finances of many sheep and goat breeders. However, coenurosis can also be controlled effectively through preventative measures, such as anthelmintic treatment of dogs and the proper disposal of intermediate host carcasses. The parasite is also zoonotic, and cases of coenurosis have been reported in humans with coenuri located in the brain, spinal cord and eyes.

**Graphical abstract:**

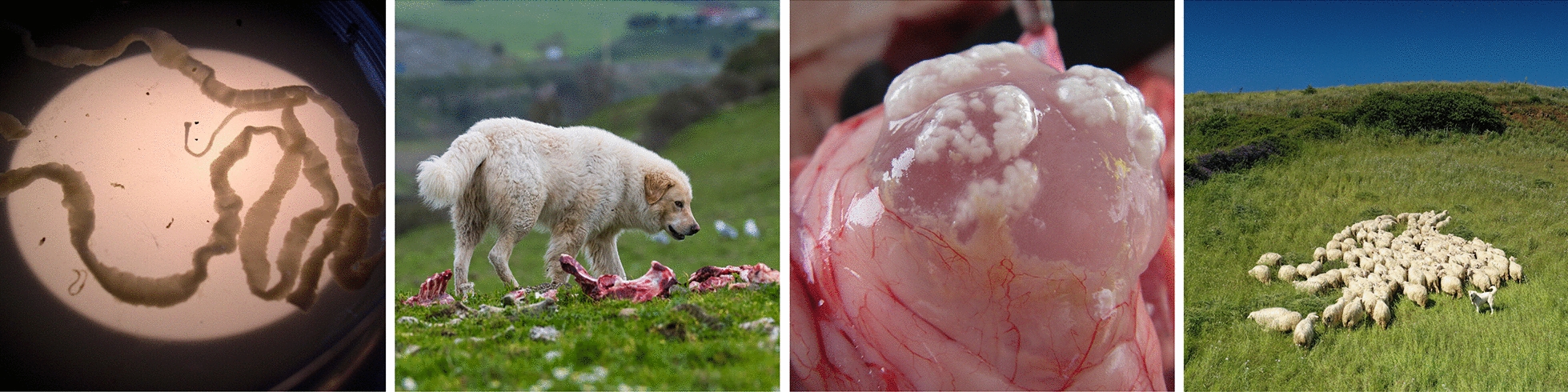

## Background

*Taenia multiceps* (Leske 1780) (larval stage *Coenurus cerebralis*), often known as sturdy or gid, is a cestode that typically affects the central nervous system (CNS) of livestock, particularly the brain and spinal cord [1]. The characteristic clinical signs of the disease were most likely reported for the first time by Hippocrates (400–375 BC), who described an excess of fluid in the brain, and considered it to be a condition causing epilepsy in sheep and goats. The earliest documented record of coenurosis dates all the way back to 1600, when an increase in cases of gid was linked to an increase in the number of sheep, and resulted in a better understanding of the parasite and the disease. Studies from 1656 and 1724 also reported the presence of water-filled sacs or bladders in sheep and cattle, and stated that the disease was a frequent cause of vertigo and death in these animals. It was not until 1780 that the cestode nature of coenurosis was established. Finally, the entire life history of *Taenia multiceps*, which then had the scientific name *Taenia coenurus*, was described experimentally in 1853 by feeding a cyst taken from an infected brain to a dog, which successfully produced the tapeworms capable of producing the coenuri seen in sheep brains. *Taenia multiceps* is so named because of the presence of multiple heads on the coenurus wall [2]. The present review covers all aspects of *T. multiceps*’ biology, including its morphology, life cycle, epidemiology and molecular characterization. Additionally, the parasite’s pathogenesis, diagnosis, therapy, economic implications, zoonotic potential, and strategies for its control are also discussed.

## Morphological description

**Fig. 1:**
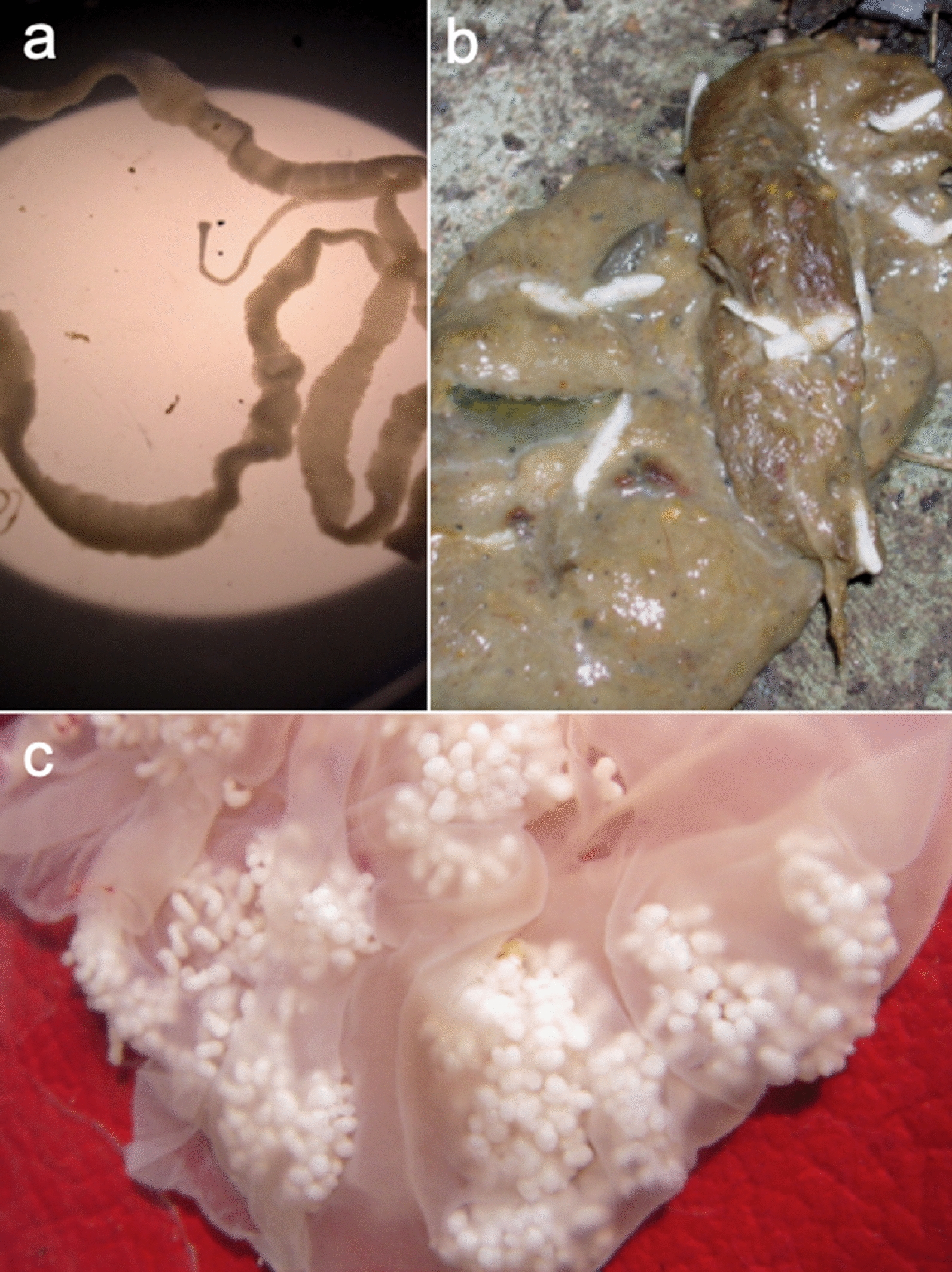
**a** Adult *Taenia multiceps* under light microscopy. **b** Proglottids of *T. multiceps* in dog feces. **c** Mature coenurus isolated from sheep; clusters of protoscoleces are clearly visible without magnification

*Taenia multiceps* is a taeniid cestode which, during its adult stage, inhabits the small intestines of domestic and wild carnivores, such as dogs, jackals, foxes and coyotes [3, 4]. The adult tapeworms range in length from 400 to 1000 mm and are 5 mm wide (Fig. 1a) [5, 6]. The scolex is 746–956 μm in diameter, with four suckers and a rostellum armed with a variable number of hooks (from 22 to 32), organized into two crowns [5–8]. The large hooks are between 157 and 177 μm long, while the small hooks measure between 98 and 136 μm in length [7]. Both immature and mature proglottids are initially wider, with a gradual increase in length towards the back of the body (Fig. 1b); they are imbricated, with an interior longitudinal muscle sheath and strongly developed transverse muscles [9, 10]. The proglottids have irregularly alternating genital pores, numerous testes (from 284 to 354) in a single anterior field, and are lateral and posterior to female organs [7, 10, 11]. The vitelline gland is simple and is situated posteriorly to the ovary, which is bilobed [10]. The proglottids are mobile and may also release eggs prior to their expulsion with the feces of the definitive host, as branches of the gravid uterus extend up to the anterior border of the proglottids [12]. When proglottids separate, the uterine branches burst explosively, releasing large number of eggs, leading to their aggregation in the feces, which tends to favor their dissemination in the environment [12]. The gravid proglottids are longer and narrower, and the uterus, with its median stem, has from nine to 25 branches on each side, each containing 32,000–37,000 eggs 30–35 μm in size that are brown in color and are surrounded by a radially striated egg shell containing the hexacanth embryo or oncosphere [13–15]. *Taenia multiceps* eggs cannot be morphologically distinguished from those of other taeniid cestodes.

*Coenurus cerebralis*, the larval stage or metacestode of *T. multiceps*, develops in the central CNS of sheep, goats, cattle, buffaloes, yaks, horses and pigs, as well as other domestic and wild ruminants [16–18]. Additionally, the parasite is also zoonotic, as evidenced by multiple cases of coenurosis in humans caused by *T. multiceps* [18–20]. The mature metacestode, *C. cerebralis*, measures 50–60 mm in diameter and appears in infected tissue as a white fluid-filled cyst encased in an adventitious membrane composed of connective tissue [15, 21]. The cyst is characterized by this thin and transparent membrane, which surrounds 400–500 protoscoleces that are invaginated from the inner membrane and are grouped into a variable number of clusters (from one to 23), which appear as white dots attached to the internal membrane (Fig. 1c) [8, 15, 17, 22]. The protoscoleces measure 281.9 μm (± 49 μm) in diameter and contain four suckers; they also have a large rostellum, 314.6 μm (± 60 μm) in length, with large and small hooks arranged in two rows [17, 23].

## Life cycle

**Fig. 2:**
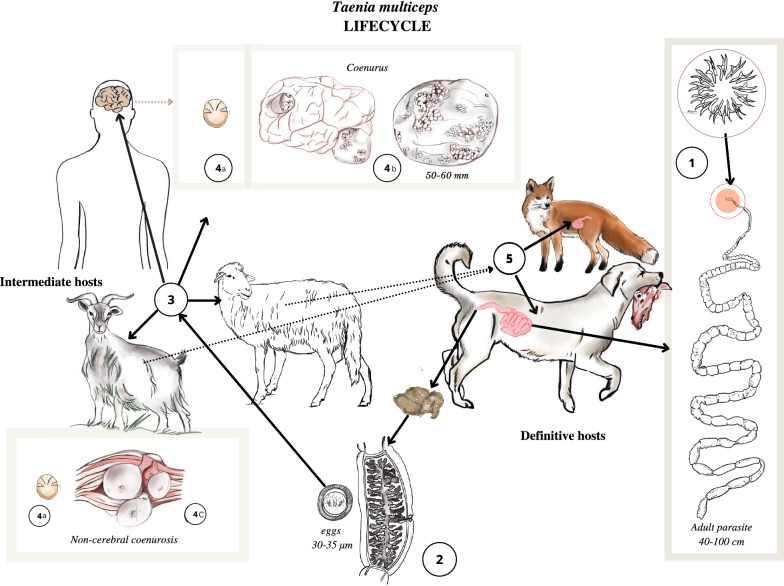
Graphical representation of the life cycle of *Taenia multiceps*. The adult *T. multiceps* (*1*) resides in the small intestine of the definitive host. Eggs or gravid proglottids are shed in the feces of the definitive host into the environment (*2*). After ingestion, the eggs hatch in the small intestine of the intermediate host (*3*) and release the oncospheres (*4a*) that penetrate the intestinal wall and migrate through the bloodstream to the central nervous system (CNS) (*4b*), and eventually, in non-cerebral forms, to subcutaneous and intramuscular tissues (*4c*). In these locations, the oncosphere encysts and develops over several months into a mature infective coenurus, *Coenurus cerebralis* (*4a*,* 4b*). Definitive hosts (*5*) are infected by ingesting the tissue of an infected intermediate host containing the mature coenurus

**Fig. 3:**
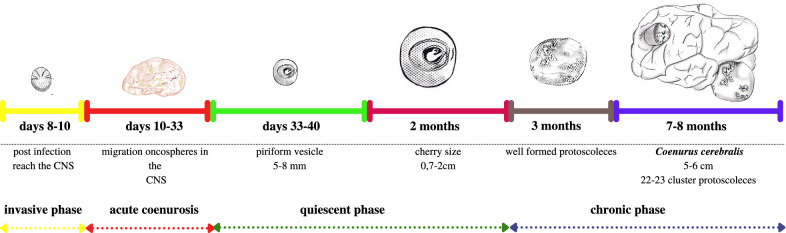
Graphical representation of the development of the *Taenia multiceps* oncosphere into a mature coenurus and the related phases of intermediate host infection

*Taenia multiceps* has a complex life cycle (Fig. 2) characterized by different stages of development and migration within its hosts [24]. After a prepatent period of 40–60 days, the definitive host releases three or four proglottids, each carrying almost 37,000 eggs, or eggs that are excreted from the proglottids prior to their shedding with the feces [1]. The eggs contaminate the environment, where they remain viable for 24 h at a high temperature, 12–15 days under dry conditions, and 3 weeks in a humid environment, or may be ingested by an intermediate host [1]. In the small intestine of an intermediate host, the oncosphere hatches from an egg and travels through the intestinal wall, primarily reaching the CNS via the bloodstream, where it encysts and matures over several months into an infective coenurus [1, 25]. The metacestode may also develop and mature in subcutaneous, intramuscular tissues and peritoneal areas as well as in organs such as the heart and lungs of sheep and goats [8, 26–32]. The oncosphere develops in the following stages: on day 8–10 post-infection (PI), it reaches the CNS and then migrates actively in the CNS from day 10 to 33 to reach its final destination; on day 40, it turns into a pyriform vesicle with just visible scoleces; after 2 months, it is the size of a cherry. Three months after infection, the cyst matures with well-formed protoscoleces, and finally, after 7–8 months, it reaches its final size of 5–6 cm in diameter (Fig. 3) [15, 21]. The life cycle is complete when the definitive host ingests the coenurus containing the mature protoscoleces [25].

## Geographical distribution and epidemiology

Coenurosis was initially reported in Europe in 1984 in Wales, where it appeared to be the most widespread neuropathological disease affecting sheep [33]. In general, low rates of infection with *T. multiceps* have been documented in other European countries, such as Ireland [34], UK [35], France [15], Switzerland [36], and Greece [37–39]. However, in Italy, high infection rates have been reported for Sardinia, with a documented prevalence of 0.35% during a large-scale epidemiological study on sheep [40], which was higher than prevalences reported for Sicily and the Apulia and Latium regions of the country [41–43]. More recent studies, on sheep [44–47], cattle and goats [17, 48], also established a higher prevalence of coenurosis in Sardinia. In addition, a recent epidemiological survey in Sardinia based on a questionnaire completed by sheep farmers estimated a prevalence of 6.3% in replacement lambs (759/12,016), with an average of 4.96 ± 9.2 sick animals per farm (A. Varcasia, unpublished data).

In Asia, enzootic coenurosis infections have been reported from India (2.9% in sheep) [49], Iran (1.7–18.6% in sheep and goats) [29, 50–53], Bangladesh (2.5% in calves and 5% in Beetal goats) [54, 55], Pakistan (0.4% in sheep and goats) [56], Japan [57] and Russia [58]. In the Middle East, cerebral coenurosis is a major endemic disease affecting small ruminants, most notably in Turkey, Egypt, Iraq, and Jordan [59, 60], with prevalences of 2.9% in Jordanian sheep [61], 22.8–23.68% in Iraqi sheep and goats [62, 63], 1.3–28.5% in Turkish sheep [64, 65], and 16.6% in Dafuri goats in Oman [59]. A recent study in the El Menoufia Province of Egypt found *C. cerebralis* in 3.03% of sheep (26.4% of clinical cases) [66], which was significantly lower than the previously reported infection rates of 18–100% [14, 67]. There have been several reports of the occurrence of coenurosis in Africa, with the lowest reported prevalences in Ethiopian sheep and goats (4–8%) [68, 69], Kenyan sheep (2.3–4.5%) [70], and Mozambique goats (14.8%) [71]. The highest reported prevalence from Africa was 42.1%, in Tanzanian sheep and goats [72]. Although no correlation was found in the available literature between the disease and different breeds of sheep and goats, there are several case reports for dwarf goats in Sardinia, Italy [44, 73].

The disease has also been reported in animals other than small ruminants, including cattle [37, 48, 74, 75], mouflon [76, 77], buffalo, horses [78, 79] and yak [80, 81]. Coenurosis in rarely seen in the tissues of small ruminants other than those of the brain or spinal cord, and has been described mainly in Asian countries, and never in Europe or the Americas. There have been several reports from Asia of non-cerebral coenurosis in small ruminants, such as in Iran [29, 31, 82], India [27, 79, 83–85], Bangladesh [86] and Pakistan [60, 87]. Outside of Asia, non-cerebral coenurosis has been reported in goats in Oman [88], Sudan [89], Namibia [90], Mozambique [71, 91], and in sheep (0.008%) and goats (0.3–1.75%) in the United Arab Emirates [26, 92, 93], where previously 16% prevalence was reported in goats [28].

The parasite is typically prevalent in rural areas, where the dog-sheep route appears to be the most important transmission pathway [94]. The disease may also potentially be spread by wild animals, such as foxes and wild ungulates (Fig. 2) [4]. Farmers can also contribute significantly to environmental contamination with this parasite by opening the skulls of diseased sheep out of curiosity or to establish their own personal diagnosis, enabling stray dogs to freely access *Coenurus* cysts or directly contaminating dogs by feeding them with infected tissue [1]. This metacestodosis is thought to be largely transmitted by herding and stray dogs because of their feeding patterns, which greatly increase the likelihood of them encountering infected intermediate host tissue [1, 95]. Foxes have long been considered important definitive hosts of *T. multiceps*, and a recent study provided clear experimental evidence that the red fox is a competent definitive host of this parasite, as it excreted proglottids with viable and infective eggs that could maintain the parasite’s life cycle [17], although a longer life cycle of the parasite has been observed in this species of fox. Foxes excrete gravid proglottids on or after the hundredth day PI (author’s personal observation). The presence of cysts in the brain of an infected sheep has been linked to a thinner skull, making it easier for predators like foxes to gain access to the infected CNS [1]; it has also been observed that foxes may access the full content of a sheep’s skull (the CNS and eventually the coenuri) via the foramen magnum without actually breaking the skull [17]. Therefore, the importance of wild canids such as foxes to the epidemiology of coenurosis should not be overlooked. Besides its broad host range (wild canids and stray dogs), the transmission of *T. multiceps* is also difficult to control, as, for example, male foxes can travel up to several kilometers during the spring mating season [1, 4, 95]. The prevention of infection in regions endemic for coenurosis relies heavily on educating farmers about sanitation and taking preventative measures (such as properly disposing of dead/butchered animals) [94]. As a preliminary step, the official slaughtering of animals infected with* T. multiceps*, as well as the proper disposal of animal skulls and offal, should be enforced in areas where this parasite is endemic, to prevent the spread of the disease to dogs and foxes [4]. A recent conservation effort for vultures in Sardinia, Italy, has been highlighted as an effective means of biologically disposing of carrion and offal through the establishment of controlled feeding stations that can be reached only by birds [96].

**Fig. 4:**
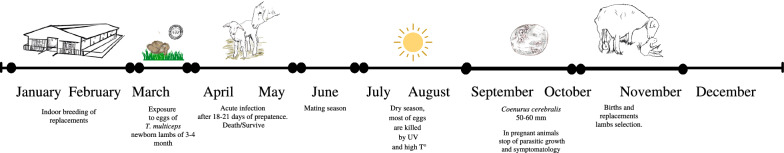
Graphical representation of the seasonality of coenurosis in Europe

Coenurosis is also related to season, with peak rates of infection reported between the spring and fall [1, 74]. Young replacement sheep are mostly exposed to the eggs of *T. multiceps* in March when the newborn lambs (3–4 months old) are let out onto pasture after the period of indoor breeding (January–February), when their immune systems and rumen activity are developing [97]. A peak of acute infections is usually observed during the spring (April–May), 10–33 days PI, and the necrotic tracks of the migrating oncospheres in the CNS can lead to the death of the infected animals. The coenurus reaches maturity after several more months, in September–October, and becomes lethal in late fall—when most of the chronic cases of the disease are observed—thus continuing the parasite’s life cycle through the infection of definitive hosts which feed on the infected carcasses. In dogs and foxes, the adult parasite develops in 40–60 days, and the elimination of mature proglottids starts at the end of winter and beginning of spring, which is when newborn replacement lambs graze, thus completing the life cycle of the parasite, which is perfectly adapted to the system of extensive sheep farming generally employed in Europe (the authors’ personal observation) (Fig. 4) [1].

## Economic losses

The economic losses associated with the prevalence of coenurosis in sheep farms are related to the category of animal most affected by the parasite, i.e. lambs and young animals bred for replacement, which are usually selected by farmers based on their genetic characteristics. Lost profit is determined by an animal’s value (replacement lambs and lambs sent to slaughter) and the losses associated with ceased production, since farmers rear the animals for 9–15 months, but they will die before providing any economic benefit (meat and milk). Loss of animal productivity, sheep fatalities and compromised genetic value for the future of the breed (chiefly for rams) are the major costs attributed to coenurosis. The slaughtering and disposal of infected animals should also be considered when estimating the total costs due to this disease.

Only a few surveys, mainly from Africa, have reported the impact of the disease on production yields and economic losses in small ruminant production systems [98–100]. The majority of these surveys were carried out in Ethiopia, where coenurosis is recognized as a primary cause (85.7%) of a diseased brain in apparently healthy slaughtered sheep and goats [101]. A recent study estimated that the financial loss due to this amounted to 3,994,272 Ethiopian birr (equivalent to US $124,821) per year [69]. A recent study undertaken by the Istituto di Servizi per il Mercato Agricolo Alimentare on the economic impact of coenurosis in Sardinia calculated an estimated loss due to lost animal value and ceased production (based on a prevalence rate of 6.3%, current costs and market prices] of €665,80/year to smaller farms and €2471/year to larger farms (A. Varcasia, unpublished data). Finally, the economic losses in sheep breeding due to coenurosis should be added to those resulting from other parasitosis, particularly metacestodosis with a similar life cycle to* Taenia multiceps*, such as *Taenia hydatigena* [102] and *Echinococcus granulosus* [94, 103].

## Molecular characterization

The significance and extent of intraspecific variation within certain *Taenia* species are unknown. A thorough understanding of cestode genetic variability and population dynamics is crucial for the effective implementation of prevention and control strategies, as they provide insight into host specialization and regional peculiarities [30, 56]. The first indication of genetic variation in *T. multiceps* was from an study on Italian sheep [77], where the genetic variants Tm1, Tm2 and Tm3 were identified using cytochrome c oxidase subunit 1 (*cox1*) and nicotinamide adenine dinucleotide dehydrogenase subunit 1 (*nad1*) analysis, with Tm1 being the most frequent variant identified. Likewise, phylogenetic analysis of *T. multiceps* sheep isolates using *cox1* gene sequences from Inner Mongolia and China revealed the presence of three genotypes with variation rates ranging from 0.25 to 0.75% [104], similar to those reported from Italy [77]. A recent study employed multiple genetic markers such as *cox1*, nicotinamide adenine dinucleotide dehydrogenase subunit 4 and cytochrome b to explore the genetic diversity of *T. multiceps* in Sichuan, China, and evidenced exceedingly low sequence variation [105]. A study that used the *cox1* gene to examine the genetic variability of *T. multiceps* in ruminants, particularly sheep, in Italy [17]found ten haplotypes (TM01-TM10) that clustered with the previously identified Tm1, Tm2 and Tm3 variants. Three distinct variants were also identified in sheep from Turkey using the *cox1* gene [106]. A recent systematic review of *T. multiceps* isolates from sheep from a range of countries [107] also identified three major haplotypes (using partial *cox1*) among the geographical populations examined, and little gene flow among the populations from Italy, despite high genetic diversity, due to the country being a peninsula. The genetic characterization of *T. multiceps* cysts from ruminants (sheep, goats and cattle) in Greece indicated five haplotypes each for *cox1* and *nad1* [108]; in the phylogenetic analysis, all the ruminant isolates clustered with previously published sequences from Italy, China and Turkey, indicating the absence of a host-specific haplotypic structure of *T. multiceps*. Low genetic diversity of *T. multiceps* was observed in Egyptian sheep, as evidenced by the presence of a low number of haplotypes of the *cox1* and *nad1* genes [66]. In contrast, *T. multiceps* isolates from sheep and goats in Iran displayed a significant degree of genetic heterogeneity, with the *cox1* gene exhibiting 11 segregation sites, resulting in seven haplotypes in sheep [3, 22]. Furthermore, a study from Pakistan [56] suggested the existence of unique *T. multiceps* haplotypes with high haplotype diversity, but low nucleotide diversity, in sheep and goats through a phylogenetic analysis that placed Pakistani and Chinese isolates in a cluster at a reasonable distance from another cluster comprising isolates from other countries.

The *cox1* and *nad1* mitochondrial genes are particularly useful for the identification of *Taenia gaigeri* and *Taenia skrjabini*, which were previously assumed to be the causal agents of non-cerebral coenurosis in goats and sheep, respectively, but are now regarded as genetic variants of *Taenia multiceps* [28, 56]. Indeed, a study carried out in the United Arab Emirates using isolates obtained from subcutaneous and muscular coenuri from goats [28] revealed significant differences from other *cox1* sequence deposited in GenBank, implying the occurrence of different genotypes or strains of *T. multiceps* in this intermediate host. The implication of *T. multiceps* in both cerebral and non-cerebral coenurosis in goats was also assessed through an experimental study in which the cysts of these two forms of coenurosis were identical to each other according to *cox1* and *nad1* sequence analysis [3]. Similarly, phylogenetic analysis of the *nad1* and *cox1* genes from cerebral and non-cerebral isolates from Indian goats indicated that they were closely related and descended from a common ancestor but were expressed differently and showed a predilection for specific sites [85]. Another study from Iran using the exonic region of enolase, *cox1* and *nad1* genes [82] established that cerebral and non-cerebral isolates from sheep and goats were 100% identical. Similar findings were reported from China using *cox1*, 12S ribosomal RNA (12S rRNA) and internal transcribed spacer regions of ribosomal DNA genes [109]. A phylogenetic assessment of isolates (*nad1*, *cox1* and 12S rRNA genes) from western Asia, Africa and Europe yielded no genetic structure with respect to cerebral and non-cerebral types, intermediate hosts or geographical location [30].

Limited data have been published on the molecular characterization of *T. multiceps* in cattle, which mostly indicated similar genetic variants of *T. multiceps* to those found in sheep and goats [17, 74]. Likewise, phylogenetic reconstructions based on 18S rRNA, *cox1* and *nad1* gene sequences showed that a *T. multiceps* yak isolate from China was closely related to *T. multiceps* isolates from other hosts and geographic locations [80].

A significant association was observed between hook length and genetic variants of *T. multiceps* using 12S rRNA nucleotide sequences, while the absence of an association between hook size and *T. multiceps* haplotypes was reported using the *cox1* gene [17, 22]. The mitochondrial *cox1* gene is one of the universally accepted markers for genetic diversity analysis [107]. Further studies using the *cox1* marker which involve more isolates from different regions and different animal species are needed to gain a clearer understanding of the genetic diversity of *T. multiceps*.

### Haplotype network of *T. multiceps*

**Fig. 5:**
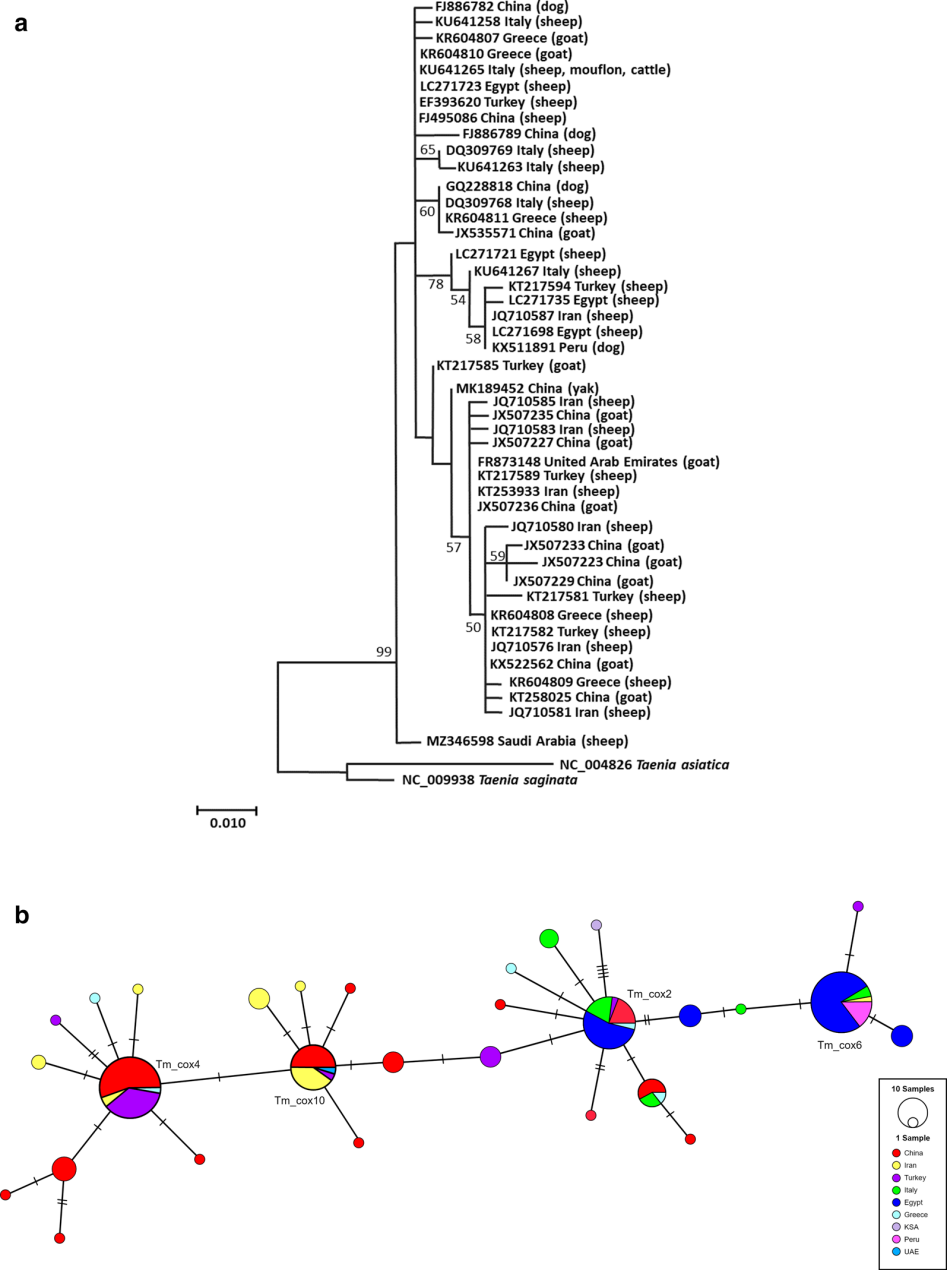
Maximum likelihood phylogenetic trees for the haplotypes obtained for partial **a** cytochrome c oxidase subunit 1 [*cox1*; 366 base pairs (bp)] and **b** nicotinamide adenine dinucleotide dehydrogenase subunit 1 (*nad1*; 471 bp) genes of *Taenia multiceps* originating from different geographical locations, using 1000 bootstrap replicates. Bootstrap values are indicated as numbers at the nodes. Bootstrap values below 50 are not shown. Intermediate hosts from which the haplotypes were identified are indicated in parentheses

**Fig. 6:**
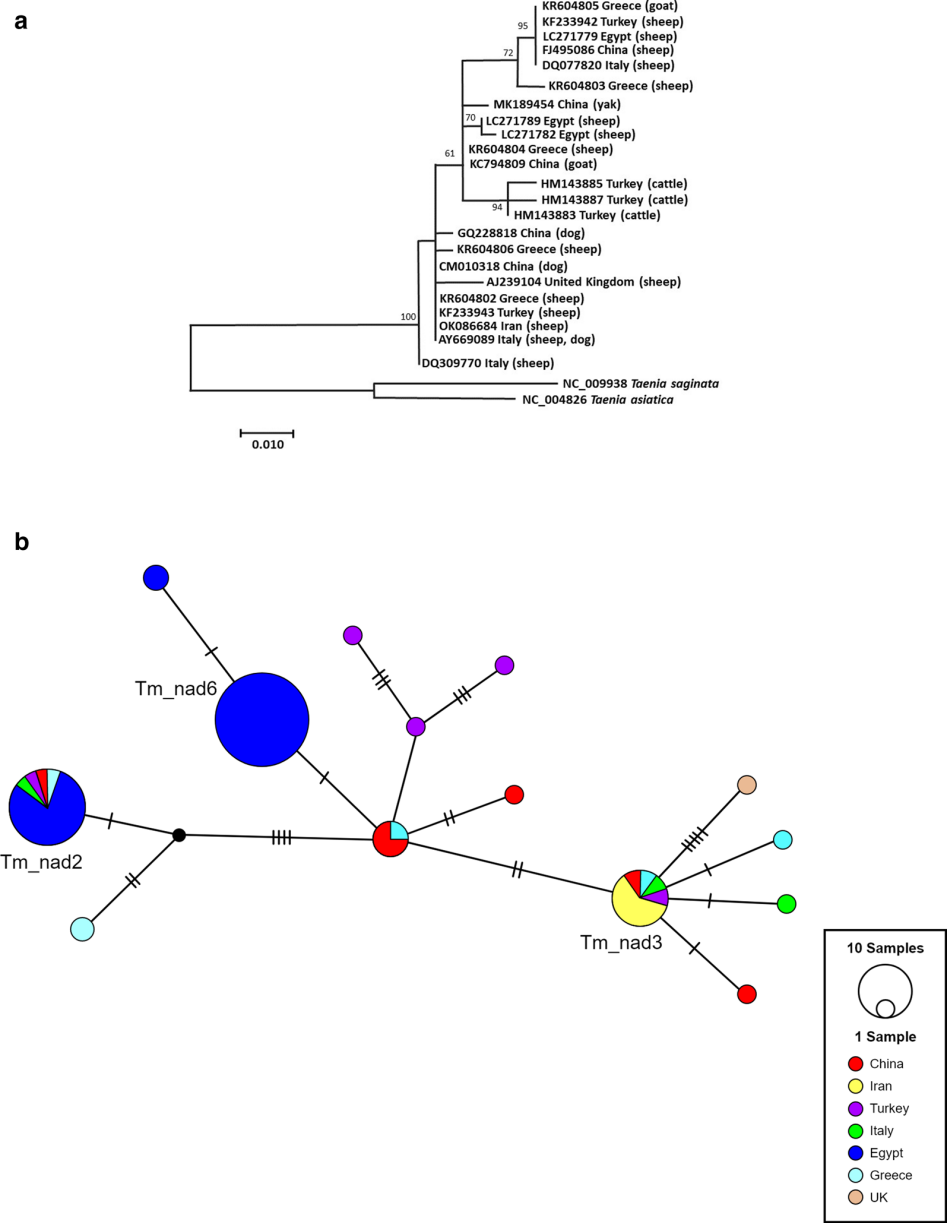
Haplotype networks reconstructed with TCS software for the partial **a** *cox1* and **b** *nad1* genes indicating the distribution of *Taenia multiceps* in different geographical populations. The number of perpendicular lines corresponds to the number of mutations between the haplotypes, and the size of the circles indicates the frequency of each haplotype. Black circles represent hypothetical haplotypes in the population

In order to understand the current genetic diversity of *T. multiceps* isolates originating from different regions of the world, nucleotide sequences of the partial *cox1* and *nad1* genes published to date in GenBank were retrieved. For both genes, the datasets were reduced according to median lengths [366 base pairs (bp) for *cox1* and 471 bp for *nad1*]. A total of 170 *cox1* and 76 *nad1* sequences were analyzed to review the extent of genetic diversification in different populations of *T. multiceps* (from China, Iran, Turkey, Egypt, Italy, Greece, Saudi Arabia, Peru, and the United Arab Emirates). High haplotype diversity was found for both *cox1* (0.881 ± 0.012) and *nad1* (0.7621 ± 0.034) genes, encompassing 31 and 14 haplotypes, respectively. Interestingly, three major haplotypes of both genes (*cox1*—Tm_cox2, Tm_cox4, Tm_cox6; *nad1*—Tm_nad2, Tm_nad3, Tm_nad4) dominated the *T. multiceps* population accounting for more than 50% prevalence among the isolates. Phylogenetic trees were drawn for both genes, in which no region-specific or host-specific patterns were observed (Figs. 5a, b; 6a, b).

## Clinical presentation and pathogenesis

Coenurosis affects sheep during their first year, and mainly lambs aged 3–4 months [1, 33]. There are rare reports of clinical signs in sheep older than 3 years, in animals with a general immunodeficient condition where they fail to produce antibodies, or in intensively bred sheep that have never been exposed to the parasite before [47].

Coenurosis in sheep is referred to as acute or chronic gid or sturdy [8, 40]. Acute coenurosis occurs as a result of the migration of oncospheres in the CNS, and the severity of the clinical signs is strictly related to the number of viable eggs ingested by the lamb, the immune status of the host (animals raised without their mothers’ colostrum are most susceptible), localization of the parasites in the CNS and inflammatory response [1, 40, 70]. The number of viable eggs ingested is crucial, as 5000 or more eggs are needed for the development of cerebral coenurosis [4, 110]. The time taken from larval hatching, migration to the brain and evidence of neurological dysfunction varies between 2 and 6 months [111].

**Fig. 7:**
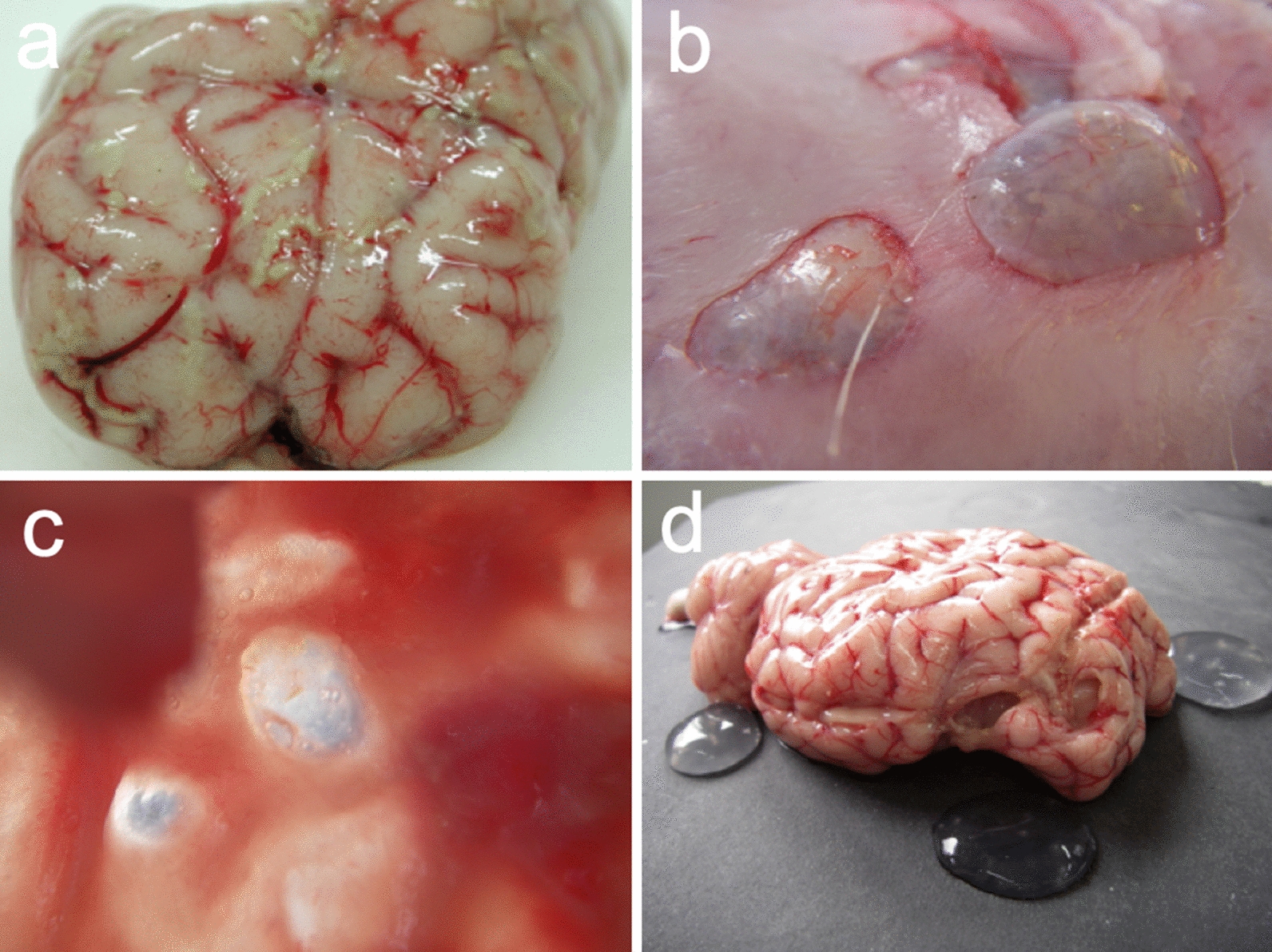
**a** Multiple linear reddish-yellow foci of purulent inflammation and necrosis indicative of parasitic larval migration during acute (gid) forms of coenurosis. **b**, **c** Thickening of the skull caused by the growth of the coenurus in the CNS in chronic forms. **d** Multiple coenuri in sheep

Acute coenurosis occurs generally 10–33 days after infection during the migration of oncospheres in the CNS; its symptoms are caused mostly by an acute inflammatory response related to a toxic and allergic reaction rather than by the mechanical action of the oncospheres, and are proportional to the number of migrating oncospheres (Fig. 7a) [1, 12]. Only 13% of lambs experimentally infected with viable eggs of *T. multiceps* showed signs of acute gid [1, 12]. Clinical signs appear within 10 days and range from mild to severe, with death occurring within 3–5 days after the onset of neurological dysfunction [111]. If the metacestode is destroyed by the host immune response, clinical recovery is complete. During necropsy, only small caseous lesions are discovered in such subjects [97].

When an animal survives acute gid (10–33 days PI), a quiescent phase lasting between 1 and 10 months is reported in which the parasite grows into a cyst; sometimes the young metacestode can be destroyed by the immune response with the complete recovery of the animal [21, 97]. This quiescent phase has also been observed in pregnant sheep, where the symptoms of coenurosis seem to disappear until the birth of the lambs, after which the neurological symptomatology reappears, causing death (A. Varcasia, unpublished data).

Chronic gid occurs in growing sheep aged 9–18 months, and is rarely reported in sheep older than 3 years [1]. Clinical signs of chronic gid are a consequence of the development of a coenurus, or coenuri, that slowly and progressively creates a local lesion in the cerebrum, cerebellum or spinal cord [40, 111]. Infected sheep usually remain isolated from the flock and show a loss of reactivity to external stimuli [40]. As the cyst increases in volume, clinical signs—depression, moving in a circle, head deviation, ataxia and blindness—become more evident [70, 112]. The neurological signs are determined by the location, number and size of the cyst(s) in the CNS [1, 70]. The affected animal tilts its head to one side and turns in a circle toward the cyst’s location. The disease has no definite clinical symptoms, but the occurrence of cerebral coenurosis in naturally or experimentally infected sheep and goats leads to a variety of neurological symptoms such as ataxia, incoordination, drowsiness, paralysis, muscle weakness, head compression, rotation, blurred vision, blindness and lack of a direct light reflex in the pupil, bruxism, poor appetite, seizures and coma [8, 113]. The majority of infected animals die of starvation within a few weeks [1, 33]. Superficial cysts may cause palpable rarefaction and pressure atrophy of the overlying part of the skull, to the extent of causing perforation (Fig. 7b, c) [16, 27].

The literature usually reports single or double cysts in infected sheep, but up to 12 cysts have been isolated in infected sheep in Sardinia (A. Scala, unpublished data), and a recent survey in Sardinia which used magnetic resonance imaging (MRI) and surgery documented that 30% of the infected sheep examined showed more than one coenurus in their brain (Fig. 7d) [4, 44]. The number of coenuri is usually negatively correlated with age, while the size of the cysts is positively correlated with this parameter [1, 114]. When the metacestode is localized in the spinal cord, it results in progressive paresis or hind leg paralysis. Usually, only these parasites develop into metacestodes which reach the CNS [112, 115], and the oncospheres that reach organs other than those of the CNS disappear and do not reach maturity.

A study carried out in Iran [116] reported the distribution of *C. cerebralis* in each compartment of the CNS in sheep and goats apart from the cerebellum, in which cysts were present only in sheep. The rate of infection in the parietal lobe of the right hemisphere was higher in goats (66.7%) than in sheep (55.1%), while the parietal lobe of the left hemisphere was similarly involved in both species (sheep, 30.6%; goats, 33.3%) [116].

Non-cerebral coenurosis in sheep is considered a rare disorder, with only a few reports dating back to the early 1900s [26] and some recently described cases [92]. In the latter, the cysts were most commonly found in the skeletal muscles such as the triceps brachii, the diaphragm, the infraspinatus muscle of the shoulder, thigh and abdominal muscles, and subcutaneous tissue of the thigh, and were also attached to the omentum [92]. No clinical evidence of non-cerebral coenurosis was recorded during the antemortem veterinary inspection of the sheep [92]. In severe cases, lameness, paresis and paralysis together with the occurrence of lumps on the skin due to the growth of the subcutaneous cysts are the major clinical manifestations of this disease [8, 51, 117, 118]. Non-cerebral forms are more common in goats, with numerous reports of cysts developing in the musculoskeletal system and subcutaneous connective tissue of goats mainly from China, Africa and the Middle East [8, 119]. Similar types of cysts have also been described in the peritoneal and pelvic cavities [118], the liver [27] and the lungs [120]. The non-cerebral location of the cysts is thought to depend on the host species, as in sheep they mature mainly in the brain, while in goats they can also grow outside of the CNS [79, 119].

In goats, non-cerebral coenurus cysts have long been called *Coenurus gaigeri*, whereas in sheep they are called *Coenurus skrjabini* [26, 79, 121]. Because the scoleces of the extra-cerebral bladder worms *Coenurus gaigeri* and *Coenurus skrjabini* exhibit the same morphological characteristics as *Coenurus cerebralis*, including shape, number and dimensions of the rostellar hooks, several authors questioned the validity of their different scientific names and considered them synonyms [7, 119, 121]. In a study carried out by Varcasia [28], molecular comparison of coenuri collected from infected goats in the United Arab Emirates with coenuri collected from sheep (Italy) and cattle (Turkey) showed that the specimens were not significantly different from each other, and that they should be considered different genotypes or strains of *T. multiceps* rather than individual species [28]. More recently, molecular evaluation of cerebral and non-cerebral coenuri from sheep and goats established that the larval stage of *Taenia multiceps* in the brain and other aberrant sites is a monophyletic species, therefore *Taenia gaigeri* in goats and *Taenia skrjabini* in sheep are not taxonomically valid [32, 82].

## Pathological findings

During the acute phase of coenurosis (10–33 days PI), multiple linear reddish-yellow foci of purulent inflammation and necrosis indicative of parasitic larval migration mostly in the cortex are visible in infected sheep brains during gross anatomo-pathological inspection (Fig. 7) [47]. Microscopic examination shows that the tracts are composed of necrotic tissue surrounded by hemorrhage and leucocyte infiltration. Eosinophils and giant cells predominate in the inflammatory reaction surrounding these tracts [111]. Fibrin-purulent meningoencephalitis and granulomatous reactions are the common histological patterns in outbreaks of acute coenurosis [47, 122]. Recently, in an atypical outbreak of acute coenurosis in sheep in Sardinia, multifocal pyogranulomatous meningoencephalitis with necrosis, macrophages, epithelioid cells, and multinucleated giant cell infiltration often surrounding degenerate parasite cysts, were observed, with rare parasitic cysts (12–15 mm wide) found in the leptomeninges [47].

In chronic coenurosis, the increased intracranial pressure (ICP) from the cyst compresses surrounding brain tissue and may result in the softening of an area of the skull; however, such changes may not occur in the bone overlying the cyst. Hydrocephalus may result from a coenurus cyst located in a ventricle or the cerebral aqueduct. Increased ICP may cause herniation of the vermis of the cerebellum through the foramen magnum, or the cerebrum may become herniated beneath the tentorium [111].

Cysts are found in the cerebral hemispheres in 90–95% of cases, where they protrude into the cerebral ventricles and near to the surface of the parietal cerebral cortex [113, 123, 124]. In a survey of sheep in Sardinia [40], most of the lesions were found in the cortex (80.6%), and mainly in the middle part (52.3%) compared to the frontal (26.1%) and occipital parts (21.6%); only 7.3% of the lesions were found in the cerebellum and 5.7% in the thalamus, and they were only sporadically found in other sites [40].

In macroscopic post-mortem inspections of the brains of infected Lori sheep, hyperemia, meningeal edema and cysts of *C. cerebralis* were seen in the ventricles along with a large number of protoscoleces [113]. Microscopic evaluation of the brain tissue typically reveals diffuse meningoencephalitis with infiltration of multinucleated inflammatory cells (eosinophils and neutrophils) in the meninges and the brain, perivascular inflammation, cerebrovascular thrombosis, necrosis, and tissue destruction [8, 113]. The cerebral tissues also exhibit cyst walls with numerous giant cells, vascular congestion surrounded by inflammatory cells such as lymphocytes and macrophages, and a cellular reaction primarily composed of numerous multinucleated giant cells and lymphocytic reactions, all of which are indicative of chronic encephalitis [123]. In addition, parts of the brain tissue may also show liquefactive necrosis due to parasitic migration. Several authors have reported liquefactive necrosis around the cerebral cysts due to degenerative changes, with satellitosis, neuronophagia, and diffuse microgliosis leading to the formation of microglial nodules [70]. The meninges of infected animals are hyperemic and edematous [8, 27, 113].

Histopathological findings in cerebral coenurosis include focal pressure atrophy, congestion, hyperemia, perivascular cuffs predominantly composed of mononuclear cells, demyelination, liquefactive degeneration and focal necrosis, besides neuronophagia, satellitosis and diffuse microgliosis, leading to the formation of microglial nodules [125–127]. The pathological lesions in hepatic coenurosis include compression of hepatic lobules, sinusoidal dilatation, vacuolar degenerative changes in the cytoplasm, karyorrhectic changes in the nucleus, and a reduction in the size of hepatocytes, leading to elongation of the cells [27, 128].

## Diagnosis

A number of factors must be taken into account in the diagnosis of cerebral coenurosis, including the age of the animal, clinical manifestations, and the results of neurological, ultrasound, and post-mortem examinations [8, 128]. The interpretation of clinical signs combined with accurate localization of the cyst using diagnostic imaging is recognized as the best diagnostic method [8, 129], although recent surveys have shown that a correlation between the clinical signs and the location of the cyst is unlikely to be of much significance [73, 130].

The careful observation of clinical signs has long been considered helpful for the approximate localization of a cyst, e.g. if the compulsive circling behavior commonly observed in sheep with chronic coenurosis manifests as circling an area of narrow diameter (1–2 m), involvement of the basal nuclei deep within the forebrain is indicated, whereas wider circles suggest a more superficial location of the cerebral cyst [111]. Interestingly, Scott [111] observed that a sheep will rotate its head towards the side where cysts are superficial and away from the side where cysts are deeper. Depression and head-pressing behavior occur when cysts are located in the frontal lobe of the cerebrum. The softening of the frontal bone, which results from a generalized increase in ICP in chronic coenurosis, may be palpable, but it is not a reliable guide for precisely locating a cyst. The presence of a cyst in one cerebral hemisphere causes loss of the menace reflex in the contralateral eye, just as blindness in the right eye indicates a lesion in the left hemisphere. More generally, unilateral proprioceptive deficits are indicative of a contralateral cerebral cyst, whereas bilateral deficits are more likely indicative of a cerebellar cyst. Deterioration of the clinical condition occurs more rapidly with a cerebellar cyst [111]. However, in light of recent findings, the correlation between clinical signs and a cyst’s location is extremely weak, especially when more than one cyst grows in the brain of a host, which was seen in about 30% of cases of coenurosis reported in Sardinia. The clinical signs of coenuri located in different sites may be similar, resulting in a very confused clinical picture [17, 44].

Several diagnostic methods have been tested over the years to determine the location of cysts, including radiography and ultrasonography, but in recent years, MRI and computed tomography (CT) have supplanted these older methods, since they allow more precise cyst localization, which is crucial for surgical removal of the cyst [34, 73, 120, 130–132]. Electroencephalography has also been used in the past to better understand the functional condition of the brain; however, it failed to provide information on the etiology or location of coenuri [133]. The use of radiography to aid in the localization of a cyst was described by Tirgari [131], but, unfortunately, interpretation of the radiographic findings is not easy. Ultrasound diagnosis is very useful for determining the location of cysts when the frontal bones of infected animals are very soft. The cysts appear as fluid-filled cavities when the transducer is placed on the soft bony part in the transverse plane in a ventral direction and then angled rostrally and caudally [129]. MRI scans can detect diffuse and focal morphological changes in cranial bones, such as a change in the shape of the frontal bone and extensive thinning of the inner cortical surfaces of the frontal, parietal, and occipital bones [73]. Typical signs of increased ICP in the brain parenchyma have been identified through MRI images, and positively linked with cyst volume by Manunta [73]. A study on neurocysticercosis in humans [73] showed that *T. multiceps* coenuri act similarly to slow-growing lesions in the brain tissue, allowing adjacent structures to adapt to the increased pressure over time. The examination of cerebrospinal fluid (CSF) is often carried out as a secondary diagnostic tool as it can be collected safely from the lumbar region under local anaesthesia from sheep of all ages [111] or during MRI [130]. There are few reports in the literature of a consistent association between an increased eosinophil concentration in the CSF and parasitic infection of the CNS [34], but examination of the CSF may be useful for ruling out other diseases characterized by a consistent intrathecal response, such as listeriosis and bacterial meningoencephalitis [111].

The other ancillary tests that may be useful for the diagnosis of suspected cases of cerebral coenurosis include determining the percentage of serum creatine isoenzyme, which usually increases when brain tissue is damaged [134]. A large number of recent studies based on serological screening through ELISA employed recombinant proteins such as Tm-HSP70, Tm-GP50, Tm-GST and Tm-HSP60, but unfortunately these tests did not provide sufficient diagnostic accuracy [135–137]. Indirect ELISA based on the recombinant antigens Tm16 and Tm18 has proven to be useful for monitoring the immune response during vaccine trials [21, 25].

### Differential diagnosis

Several diseases should be considered in the differential diagnosis of cerebral coenurosis, such as listeriosis, nasal bots syndrome, louping ill, scrapie, sarcocystosis, polioencephalomalacia and cerebral echinococcosis [8, 27, 70, 111]. The age of an infected animal may aid diagnosis, since the symptoms of cerebral coenurosis typically manifest in young animals; however, immunocompromised animals as well as animals raised in isolation may develop coenurosis when adult [1, 47]. In Sardinia, a study on 178 sheep with neurological symptoms revealed the presence of coenurosis in 6.2% of all cases, whereas scrapie was detected in 32% of cases, polioencephalomalacia in 14%, suspected intoxication by *Cistus* species in 14%, *Listeria monocytogenes* infection in 4.5%, and focal symmetrical encephalomalacia in 3.4% [45]. A study in Iran demonstrated the concurrent occurrence of *C. cerebralis* and *L. monocytogenes* in a sheep [138].

The sheep nasal bot *Oestrus ovis* (Diptera: Oestridae) may reach the brain during its migration, and an infected sheep may present similar neurological signs to those reported in acute coenurosis, such as depression, circling, head pressing, compulsive walking, blindness, and ataxia, which have given this syndrome its name, ‘false gid’; however, these symptoms are often accompanied by sneezing and nasal discharge in sheep nasal bot, which allow differential diagnosis [139]. Scrapie typically affects sheep over the age of 3 years; other diseases, such as polioencephalomalacia, cause diffuse bilateral cerebral signs; listeriosis results in multiple unilateral cranial nerve deficits; and focal symmetrical encephalomalacia results in quick death [111]. Brain abscess should also be included in the differential diagnosis list, but the clinical signs of this tend to remain relatively static and do not deteriorate as in chronic coenurosis [111]. In cases where a coenurus cyst is located within the spinal cord, the clinical presentation (upper motor neuron signs in the hind legs) is similar to that of vertebral empyema and sarcocystosis, and a successful diagnosis may not always be achieved [111].

## Immune response and vaccination

Most of the naturally occurring cases of clinical coenurosis in sheep have been observed in young animals [40], suggesting the existence of an age-related resistance to *T. multiceps* infection in sheep, although the mechanism by which this may occur is unknown. The size and fertility of the parasites detected at necropsy in numerous studies suggested that the lambs come into contact with the parasite at a young age, and hence age-related resistance to infection could potentially be attributed to changes in physiological factors associated with a lamb’s growth. Furthermore, not all exposures to *T. multiceps* result in the development of a mature coenurus, but they do, nevertheless, result in the development of immunity [21].

The first attempt to develop a vaccine against *T. multiceps* employed an oncosphere secretory antigen, which demonstrated promising signs of protection in young sheep against the larval stage of *T. multiceps* [140]. A successful vaccine trial based on homologues of the 16k and 18k (Tm16 and Tm18, respectively) families of oncosphere antigens induced a significant level of protection in sheep against an experimental challenge infection with *T. multiceps*; five of nine control animals died following the experimental challenge, whereas none of the 20 vaccinated animals died [25]. In addition, the effects of vaccination appeared to depend on the site where the cysts were located, with a lower proportion of cysts detected in the parieto-occipital region of the brain in vaccinated sheep compared with the control animals.

The first successful field test of practical vaccination against *T. multiceps* was conducted in Sardinia on six sheep farms with a known history of lamb mortality due to coenurosis [21]. A total of 632 lambs aged 10–12 weeks were selected, out of which 208 lambs received a vaccine containing recombinant protein Tm18 of *T. multiceps* together with Quil A as an adjuvant through two subcutaneous injections given approximately 1 month apart. After a period of more than 40 months from the beginning of the field trial, 33 episodes of cerebral coenurosis were identified on the monitored farms, comprising 32 cases in control sheep and only one case in a vaccinated animal (*X*^2^ = 14.08, *P* < 0.001). Serological analysis of 60 vaccinated animals tested for their antibody responses found that 98% of them responded to the vaccine with the production of specific serum antibodies [21].

A vaccine for coenurosis could significantly reduce the financial losses incurred by sheep farmers in regions endemic for coenurosis, especially Sardinia, where this parasitosis is hyperendemic; a vaccine against coenurosis could also potentially be combined with one against *Echinococcus granulosus*, which causes cystic echinococcosis, for the control of both of these metacestodosis [21].

## Therapy and control measures

The control of coenurosis is difficult and has been unsatisfactory to date. Once the clinical syndrome of coenurosis has been established, the prognosis is bad as the outcome is usually death [141]. Although the surgical treatment of coenurosis is frequently successful, the use of surgery in animals, especially in small ruminants, is limited and restricted to economically viable and genetically superior and valued animals, and it is not practiced commonly under field conditions [142].

Effective anthelmintic treatment of coenurosis in sheep with praziquantel at a dose of 50–100 mg/kg body weight was first applied by Bankov in 1977 and then by Verster in 1982, and resulted in the prevention of the formation of cysts in the brain [143 and references therein]. The efficacy of antiparasitic drugs such as albendazole, fenbendazole, as well as praziquantel, against cerebral coenurosis has been supported by numerous studies [58, 144]. A more recent study by Ghazaei [142] tested albendazole, fenbendazole, praziquantel, and a combination of praziquantel plus fenbendazole against coenurosis, and demonstrated high efficacy of albendazole at a dosage of 25 mg/kg for 6 days. Albendazole penetrates well into the CSF, which increases its efficacy [145]. Ghazaei [142] also demonstrated the efficacy of the co-administration of fenbendazole and praziquantel against coenurosis; however, to fully exploit the potential of praziquantel, a full dose of the drug is recommended (100 mg/kg) in combination with a full dose of fenbendazole. In all four treatment groups, a reduction in neurological symptoms was reported between the second and fifth days of anthelmintic treatment [142]. Due to the short duration of treatment with albendazole, for the two successful protocols chemotherapy could be used only during the migration stages of the parasite, because once the coenurus is fully developed its rupture after treatment can be very dangerous. To date, no specific therapeutic strategies are available for non-cerebral coenurosis [8].

In small ruminants, surgery is still the most effective method for the treatment of cerebral coenurosis, and has a success rate of more than 70% [129, 146, 147], although there are occasional reports of failed attempts to remove cysts surgically [16]. The percentage efficacy of surgical procedures can be as high as 90% when the brain and skull are initially examined by MRI [8, 44]. After the cyst has been precisely located by MRI, it can be grasped through a 0.5-cm-diameter hole, followed by aspiration of the cystic fluid and removal of the collapsed cyst through gentle traction of its wall. The neurological outcome was considered excellent from the 8th to the 30th day after surgical removal of the cyst, suggesting that the symptomatology may have been mostly due to a diffuse or localized increase in ICP [44, 130]. Despite the high recovery rate of sheep following the removal of cerebral cysts, the poor clinical condition of the animals when examined by a veterinary surgeon, and their low financial value in comparison to the cost of general anaesthesia and cranial surgery, may prompt most farmers to slaughter those sheep that are fit for market and euthanize those in poorer condition [111].

The most effective means of controlling coenurosis, however, are preventative strategies. The control of coenurosis can be achieved through the regular anthelmintic treatment of farm dogs with an effective taeniacide (i.e. praziquantel at 5 mg/kg body weight) at 6- to 8-week intervals and the proper disposal of sheep carcasses to prevent scavenging by herding and stray dogs [111, 113] as well as foxes [17].

## Zoonotic aspects

Coenurosis is of major zoonotic concern as it can cause life-threatening complications in humans [79, 148]. Individuals, often children, become intermediate hosts when they accidentally acquire the infection by ingesting eggs upon exposure to the feces of definitive hosts [20]. After the ingestion of the eggs, oncospheres are released into the host’s gut, where they puncture the intestinal wall and move through the bloodstream to the host’s target organs, typically the brain, spinal cord, and eyes [20, 148–150], and occasionally lodge in intramuscular tissue, subcutaneous tissue and pericardial and abdominal cavities [151]. Transmission may also occur via a conventional foodborne pathway as a result of the ingestion of eggs from unwashed infected vegetables, fruits, or berries [152]. Human infection rates within endemic areas can vary significantly, for example, between urban and rural areas and within larger endemic areas, due to various sociocultural and economic factors.

Human coenurosis was first described by Brumpt in 1913, and was long considered an extremely rare disease [148]. However, to date, approximately 100 human cases have been reported around the world, mostly in sheep-farming regions of Africa [151, 153], Europe [154–158], Asia [159], and the Americas [148, 160–163].

The clinical signs of coenurosis in humans may vary according to the anatomical region(s) in which the larvae are lodged [148]. The larvae typically lodge in the CNS, causing a variety of symptoms including seizures, headaches, vomiting, and papilledema. Additionally, cranial nerve palsy, hemiplegia, or paraplegia may develop [156, 157]. Arachnoiditis and ependymitis may also occur as a result of involvement of the CSF [163]. Some cases with visual impairment and involvement of the trigeminal nerve have also been described [159]. The parasite may also manifest as a subcutaneous cyst, paravertebral mass, or an intraperitoneal infection, resulting in obstructive jaundice and extrinsic biliary constriction [148]. A case of ocular coenurosis was reported in Nigeria [164]. Coenurosis may also present as giant cysts in humans, which are frequently misdiagnosed as giant cysticercal cysts or hydatid cysts, and hence the disease goes undiagnosed [159].

## Conclusions

Coenurosis caused by *T. multiceps*, like other well-known metacestodoses, is a significant health concern that can lead to notable economic losses in small ruminant breeding and also constitutes a non-negligible zoonotic risk. Thus, it is appropriate to conduct further research on chemical prophylactic protocols for coenurosis (vaccines and drugs) and alternative strategies for its control, such as the prevention of infection in definitive hosts. The valorization of sheep products (and goat products) could also help to promote effective measures against this metacestodosis.

## Data Availability

The datasets used and/or analyzed during the current study are available from the corresponding author on reasonable request.

## Abbreviations

CNS: Central nervous system
*cox1*: Cytochrome c oxidase subunit 1
CSF: Cerebrospinal fluid
ICP: Intracranial pressure
MRI: Magnetic resonance imaging
*nad1*: Nicotinamide adenine dinucleotide dehydrogenase subunit 1
